# Different effects of cardiometabolic syndrome on brain age in relation to gender and ethnicity

**DOI:** 10.1186/s13195-023-01215-8

**Published:** 2023-03-30

**Authors:** Sung Hoon Kang, Mengting Liu, Gilsoon Park, Sharon Y. Kim, Hyejoo Lee, William Matloff, Lu Zhao, Heejin Yoo, Jun Pyo Kim, Hyemin Jang, Hee Jin Kim, Neda Jahanshad, Kyumgmi Oh, Seong-Beom Koh, Duk L. Na, John Gallacher, Rebecca F. Gottesman, Sang Won Seo, Hosung Kim

**Affiliations:** 1 Departments of Neurology, Samsung Medical Center, Sungkyunkwan University School of Medicine, Seoul, South Korea; 2 Department of Neurology, Korea University Guro Hospital, Korea University College of Medicine, Seoul, South Korea; 3 School of Biomedical Engineering, Sun Yat-Sen University, Shenzhen, China; 4 Keck School of Medicine of University of Southern California, USC Steven Neuroimaging and Informatics Institute, Los Angeles, CA 90033 USA; 5 Department of Psychiatry, University of Oxford, Oxford, UK; 6 National Institute of Neurological Disorders and Stroke Intramural Research Program, National Institutes of Health, Bethesda, MD USA; 7 Department of Digital Health, SAIHST, Sungkyunkwan University, Seoul, South Korea; 8 Department of Health Sciences and Technology, SAIHST, Sungkyunkwan University, Seoul, South Korea; 9 Alzheimer’s Disease Convergence Research Center, Samsung Medical Center, Seoul, South Korea; 10 Department of Intelligent Precision Healthcare Convergence, Sungkyunkwan University, Suwon, South Korea

**Keywords:** Brain age, Cardiometabolic syndrome, Gender, Ethnicity

## Abstract

**Background:**

A growing body of evidence shows differences in the prevalence of cardiometabolic syndrome (CMS) and dementia based on gender and ethnicity. However, there is a paucity of information about ethnic- and gender-specific CMS effects on brain age. We investigated the different effects of CMS on brain age by gender in Korean and British cognitively unimpaired (CU) populations. We also determined whether the gender-specific difference in the effects of CMS on brain age changes depending on ethnicity.

**Methods:**

These analyses used de-identified, cross-sectional data on CU populations from Korea and United Kingdom (UK) that underwent brain MRI. After propensity score matching to balance the age and gender between the Korean and UK populations, 5759 Korean individuals (3042 males and 2717 females) and 9903 individuals from the UK (4736 males and 5167 females) were included in this study. Brain age index (BAI), calculated by the difference between the predicted brain age by the algorithm and the chronological age, was considered as main outcome and presence of CMS, including type 2 diabetes mellitus (T2DM), hypertension, obesity, and underweight was considered as a predictor. Gender (males and females) and ethnicity (Korean and UK) were considered as effect modifiers.

**Results:**

The presence of T2DM and hypertension was associated with a higher BAI regardless of gender and ethnicity (*p* < 0.001), except for hypertension in Korean males (*p* = 0.309). Among Koreans, there were interaction effects of gender and the presence of T2DM (*p* for T2DM*gender = 0.035) and hypertension (*p* for hypertension*gender = 0.046) on BAI in Koreans, suggesting that T2DM and hypertension are each associated with a higher BAI in females than in males. In contrast, among individuals from the UK, there were no differences in the effects of T2DM (*p* for T2DM*gender = 0.098) and hypertension (*p* for hypertension*gender = 0.203) on BAI between males and females.

**Conclusions:**

Our results highlight gender and ethnic differences as important factors in mediating the effects of CMS on brain age. Furthermore, these results suggest that ethnic- and gender-specific prevention strategies may be needed to protect against accelerated brain aging.

## Background

Aging is an important risk factor for cognitive impairment and dementia [1]. As aging progresses, brain atrophy also occurs at a mean volume reduction rate of 0.5% per year after the age of 40 [2, 3]. Age-related brain atrophy is referred to as the brain age. Cardiometabolic syndrome (CMS), syndrome X, metabolic syndrome, and cardiometabolic dysfunction, composed of type 2 diabetes mellitus (T2DM), hypertension, and obesity, are critical modifiable risk factors for cognitive impairment. There is also a growing body of evidence that CMS has deleterious effects on brain atrophy [4] even in non-demented population [5, 6]. Previous studies have suggested that CMS may accelerate brain aging.

Several cross-sectional studies have shown differences in brain volume between males and females in cognitively unimpaired (CU) populations [7–9]. Previously, changes in brain age or atrophy were shown to occur differently depending on gender [3, 10, 11]. Additionally, previous studies based on Hispanic or Korean populations suggested that CMS-associated brain atrophy was more extensive or prominent in females than in males [6, 10]. However, considering the differences in the prevalence of CMS and dementia between Korean and European populations, it would be reasonable to hypothesize that there might be a difference in the gender-specific relationship between CMS and brain age between Koreans and Europeans.

Previous studies have analyzed various morphological features on brain magnetic resonance imaging (MRI), including cortical thickness [12], regional gray matter volume [11], and white matter hyperintensity [13] and integrity [14], and investigated the impact of CMS on brain structure in aging populations. Recently, various machine learning approaches have been developed to begin accurate prediction of brain age using the aforementioned brain imaging features [15–18] and provide a new metric called brain age index (BAI) to measure how old the brain age is compared to the chorological age at MRI scan. The difference between the predicted brain age using a deep learning-based algorithm and the chronological age is called the BAI, which explains how much older or younger an individual brain appears compared to the current age. A positive BAI is a novel marker of an older brain and has been shown to predict compromised brain health [19], earlier mortality [20], and cognitive impairment [21, 22].

In the present study, we investigated the different effects of CMS on BAI with respect to the sex of CU populations from Korea and United Kingdom (UK). Next, we determined whether CMS affects gender-specific BAI differently according to ethnicity. Considering that there are differences in incidence of CMS and cortical atrophy by gender and ethnicity, we hypothesized that there might be differences in the effects of CMS on the BAI in relation to gender and ethnicity.

## Methods

### Study populations

We enrolled CU participants aged ≥ 45 years from the Health Promotion Center of Samsung Medical Center (Seoul, Korea) who underwent a comprehensive health screening exam from September 1, 2008, to October 31, 2019. A total of 8227 eligible candidates underwent a full medical examination, which included cognitive assessment and 3.0-Tesla MRI, including high-resolution T1-weighted MRI, as part of a standard screening for dementia. The medical examination procedure used for the participants has been previously described [23]. We excluded participants who had any of the following conditions: 728 participants with missing data on years of education or Mini-Mental State Examination (MMSE) score [24]; 509 participants with significant cognitive impairment defined by MMSE scores below the 16th percentile in age-, gender-, and education-matched norms or through an interview conducted by a qualified neurologist; 312 participants with severe cerebral white matter hyperintensities (deep white matter ≥ 2.5 cm and caps or band ≥ 1.0 cm) or structural lesions such as territorial infarction, lobar hemorrhage, brain tumor, and hydrocephalus; 542 participants with missing information on DM, hypertension, or body mass index (BMI); and 377 participants with unreliable analyses of cortical thickness due to head motion, blurred MRI, inadequate registration to a standardized stereotaxic space, misclassification of tissue type, or inexact surface extraction. Finally, 5759 participants (3042 males and 2717 females) were included in this study.

Similar data for people of British ancestry was obtained from the UK Biobank (UKB, http://www.ukbiobank.ac.uk), a population-based prospective cohort study of approximately 500,000 people in the UK [https://journals.plos.org/plosmedicine/article?id=10.1371/journal.pmed.1001779, https://www.nature.com/articles/s41586-018-0579-z]. Of these participants, approximately 40,000 attended an additional visit during which MRI brain imaging data was collected in addition to other health-related data [https://www.nature.com/articles/s41467-020-15948-9]. We included non-Hispanic White adults only in the present study. We excluded participants with a self-reported or hospital record-based history of dementia, Parkinson’s disease, or other central nervous system-related diseases. Finally, 9903 (4736 males and 5167 females) UKB participants were included after applying the inclusion/exclusion criteria and after random selection of a smaller subset of participants for brain imaging data processing.

The institutional review board of the Samsung Medical Center approved this study and adhered to the principles of the Declaration of Helsinki. Written informed consent was obtained from all participants in the Health Promotion Center of Samsung Medical Center. Anonymous and deidentified data from the UKB was used for analysis and, therefore, the present study was exempted from obtaining informed consent.

### Measurement of cardiometabolic syndrome

For populations from the Health Promotion Center at the Samsung Medical Center, a health screening exam was conducted by a well-trained medical professional using standardized protocols. Baseline workup included blood tests (complete blood cell count, liver/kidney/thyroid function test, and tumor markers), urine analyses, abdominal sonography, chest radiography, electrocardiogram, pulmonary function test, and gastroduodenoscopy. We classified each CMS component using the following criteria: T2DM was defined as a diagnostic history of T2DM or current use of any anti-diabetic medication; hypertension was defined as a diagnostic history of hypertension or current use of any antihypertensive medication; obesity and underweight were defined using the cut-off for BMI calculated by weight (kilograms)/height (meters) squared at the first visit. According to a previous study [10], populations with BMI < 18.5 kg/m^2^ were labeled as underweight, and those with BMI ≥ 27.5 kg/m^2^ were labeled as obese.

For populations from the UKB, the classification of T2DM, hypertension, and obesity was determined based on a combination of a touchscreen-based questionnaire, a verbal interview, and linked hospital records. Specifically, T2DM was defined as either self-reported T2DM, a doctor’s diagnosis of T2DM (Data Field 2443), patients who were taking insulin (Data Fields 6177, 6153), or a hospital data-linked record of an individual with a diagnosis of T2DM. Hypertension was defined as either self-reported hypertension (Data-Field 20,002) or a hospital data-linked record of having a primary or secondary diagnosis of hypertension (Data-Fields 41,202, 41,204, 41,203, and 41,205). BMI was calculated using weight and height measurements in the same way as the Samsung Medical Center data. Populations with a BMI < 18.5 kg/m^2^ were categorized as underweight, whereas those with BMI ≥ 35 kg/m^2^ were categorized as obese [25].

### Acquisition of brain MRI

All Korean populations underwent a 3D volumetric brain MRI scan. An Achieva 3.0-Tesla MRI scanner (Philips, Best, the Netherlands) was used to acquire 3D T1 Turbo Field echo (TFE) MRI data using the following imaging parameters: sagittal slice thickness, 1.0 mm with 50% overlap; no gap; repetition time of 9.9 ms; echo time of 4.6 ms; flip angle of 8; and matrix size of 240 × 240 pixels reconstructed to 480 × 480 over a field view of 240 mm.

In the UKB populations, brain MRI scans were obtained at one of the three assessment sites using a 3.0 Tesla Siemens Skyra MRI Scanner. Among the six brain imaging modalities acquired was a T1-weighted, sagittal 3D magnetization prepared rapid gradient echo (MPRAGE) scan. The following imaging parameters were used in this T1-weighted acquisition: inversion time of 880 ms; repetition time of 2000 ms; 1 × 1 × 1 mm^3^ voxel size; 208 × 256 × 256 matrix size; and SENSE factor (R) of 2.0 [Miller, 2016: https://www.nature.com/articles/nn.4393].

### Image processing and cortical surface extraction

T1-weighted MRI scans from the Health Promotion Center in Korea and the UKB were used to reconstruct the inner and outer cortical boundaries using the CIVET pipeline developed at the Montreal Neurological Institute (http://www.bic.mni.mcgill.ca/ServicesSoftware/CIVET). Cortical morphology was quantitatively characterized by measuring cortical thickness, sulcal depth, and gray/white intensity ratio [26] on the cortical surface at 81,924 vertices (163,840 polygons). These features were further resampled to the surface template using the transformation obtained in the surface registration to allow for inter-subject comparisons.

### Development of prediction model for relative brain age

**Fig. 1:**
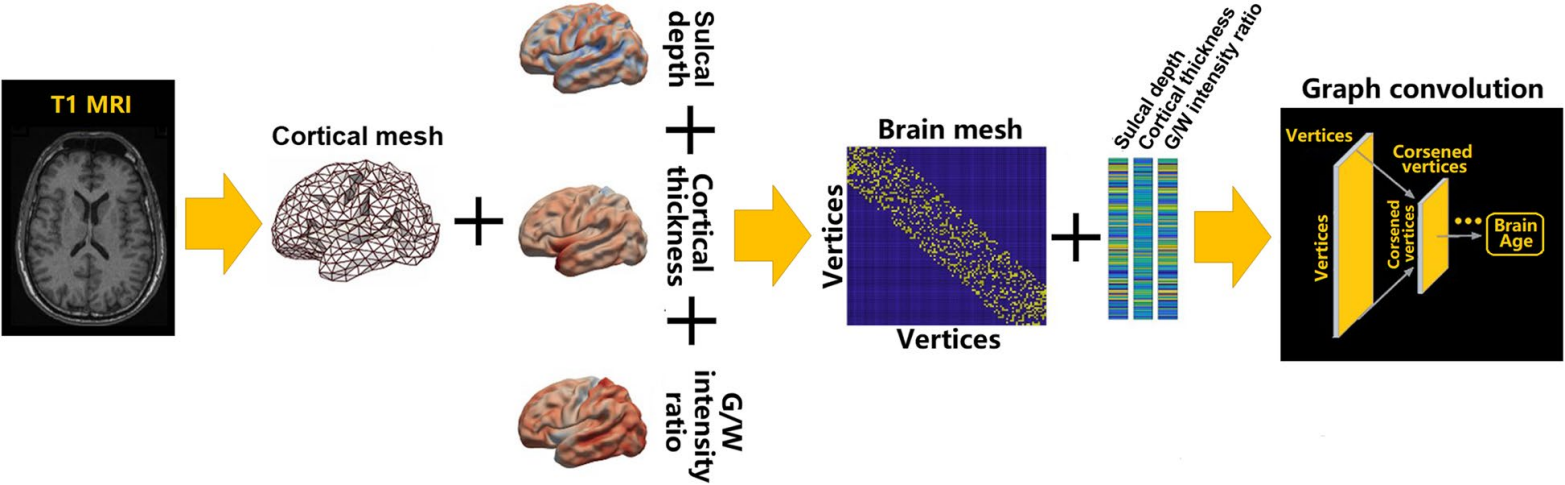
The graph-based convolutional network for brain age prediction

As illustrated in Fig. 1, we did not use topology-varying surfaces because of the nature of the graph convolutional networks (GCN) model used in this study. Rather, we considered the cortical morphological changes that occur in relation to brain size and gyrification using cortical thickness, volume, and sulcal depth. The GCN employed in our study requires identical graph/mesh structures for all individual inputs, whereas the features of the nodes/vertices can vary. Another advantage of the topology-kept surface model is that surface nodes are registered across all individuals such that anatomical information is shared.

### Brain age index

After calculating the predicted brain age for each subject, we further calculated a metric that reflected the subject's relative brain health status, called the BAI. BAI was initially measured by subtracting the true brain age from the predicted brain age [27]. Due to regression dilution [28], however, it is also possible that regression models bias the predicted brain age toward the mean, underestimating the age of older subjects and overestimating the age of younger subjects [29]. When deriving the BAI, this bias must be corrected using a strategy introduced in other studies [15, 28]. We defined the new BAI as the difference between the individual BAI and the expected BAI (measurement fitted over the entire sample set using the regression model and leave-one-out cross-validation). The BAI was corrected such that the BAIs of the whole dataset analyzed became unbiased across all age ranges.

### Propensity score matching

Propensity score matching was performed to minimize the differences in the demographics and cardiometabolic factors between the UK and KOR participants. The propensity score was obtained using multivariable logistic regression based on age, gender, T2DM, hypertension, and obesity. A total of 5541 KOR participants were matched with 9903 UK participants based on propensity scores using the 1:2 nearest-neighbor matching algorithm with caliper of 0.1. A good balance was achieved between the KOR and UK participants, with all standardized mean differences (age, gender, T2DM, hypertension, and obesity) below 0.1 after matching.

### Statistical analysis

Independent *t*-tests and chi-squared tests were used to compare continuous and categorical variables, respectively.

To explore the association between the presence of each CMS component and brain age in females and males among the Korean and UK populations, we performed a linear regression analysis with the presence of T2DM, hypertension, obesity, and underweight as covariates. To assess whether the association between the presence of each CMS component and brain age might differ by gender in the Korean and the UK populations, we performed linear regression analyses by adding each two-way interaction term (the presence of each CMS component*gender) to covariates in Korean and UK populations after controlling for the other CMS components. To assess whether the association between the presence of each CMS component and brain age might differ by gender and ethnicity, we performed linear regression analyses with the addition of each three-way interaction term (the presence of each CMS component*gender*ethnicity) to covariates after controlling for the other CMS components.

False discovery rate (FDR) correction was conducted for all statistical analyses to control for *p*-values, and *q*-values were obtained after FDR correction. All reported *p*-values and *q*-values were two-sided and the significance level was set at 0.05. All analyses were performed using R version 4.3.0 (Institute for Statistics and Mathematics, Vienna, Austria; www.R-project.org).

## Results

### Demographics of cognitively unimpaired populations in the UK and Korea

After propensity score matching, the demographic characteristics of the two ethnic datasets were similar (Table 1). Among the 5541 Korean populations, there were 2599 (46.9%) females and 2942 (53.1%) males. Among the 9903 UK populations, there were 5167 (52.2%) females and 4736 (47.8%) males. There were some differences in mean age (64.0 and 63.6 years, *p* < 0.001), female ratio (46.9 and 52.2%, *p* < 0.001), and the presence of T2DM (17.3 and 9.8%, *p* < 0.001), hypertension (42.7% and 40.6%, *p* = 0.011), obesity (10.7 and 7.9%, *p* < 0.001), and underweight (1.8 and 0.6%, *p* < 0.001) between Koreans and participants from the UK.

**Table 1:** Demographics of populations from UK Biobank and Health Promotion Center in Korea

Variables	Korea			UK			*p*-value
	**Females (** ***n*** ** = 2599)**	**Males (** ***n*** ** = 2942)**	**Total (** ***n*** ** = 5541)**	**Females (** ***n*** ** = 5167)**	**Males (** ***n*** ** = 4736)**	**Total (** ***n*** ** = 9903)**	
Age (years)^a^	63.2 ± 6.9	64.7 ± 6.5	64.0 ± 6.7	63.4 ± 7.1	63.8 ± 7.4	63.6 ± 7.2	0.007
Hypertension (*n*, %)^b^	965 (37.1%)	1402 (47.7%)	2367 (42.7%)	1813 (35.1%)	2208 (46.6%)	4021 (40.6%)	0.1
T2DM (*n*, %)^b^	273 (10.5%)	683 (23.2%)	956 (17.3%)	365 (7.1%)	602 (12.7%)	967 (9.8%)	< 0.001
BMI (kg/m^2^)^a^	23.5 ± 2.9	24.5 ± 2.6	24.0 ± 2.8	26.8 ± 5.2	27.7 ± 4.4	27.2 ± 4.9	< 0.001
Obesity (*n*, %)	227 (8.7%)	336 (12.4%)	593 (10.7%)	450 (8.7%)	336 (7.1%)	786 (7.9%)	< 0.001
Underweight (*n*, %)^b^	70 (2.7%)	32 (1.1%)	102 (1.8%)	49 (0.9%)	7 (0.1%)	56 (0.6%)	< 0.001

Propensity score matching was performed to balance the age and gender between the Korean and UK populations, and 9903 out of 17,791 populations in the UK and 5541 out of 5759 populations in Korea were selected for the present study

Abbreviations: *BMI*, body mass index; *T2DM*, type2 diabetes mellitus; *UK*, United Kingdom

^a^Distribution of age and BMI was compared between the populations of UK and Korea using independent *t* tests

^b^Distribution of education level and presence of hypertension, T2DM, obesity, and underweight between the populations of UK and Korea were tested using the chi-squared test

### Effects of cardiometabolic syndrome components on brain age index

**Fig. 2:**
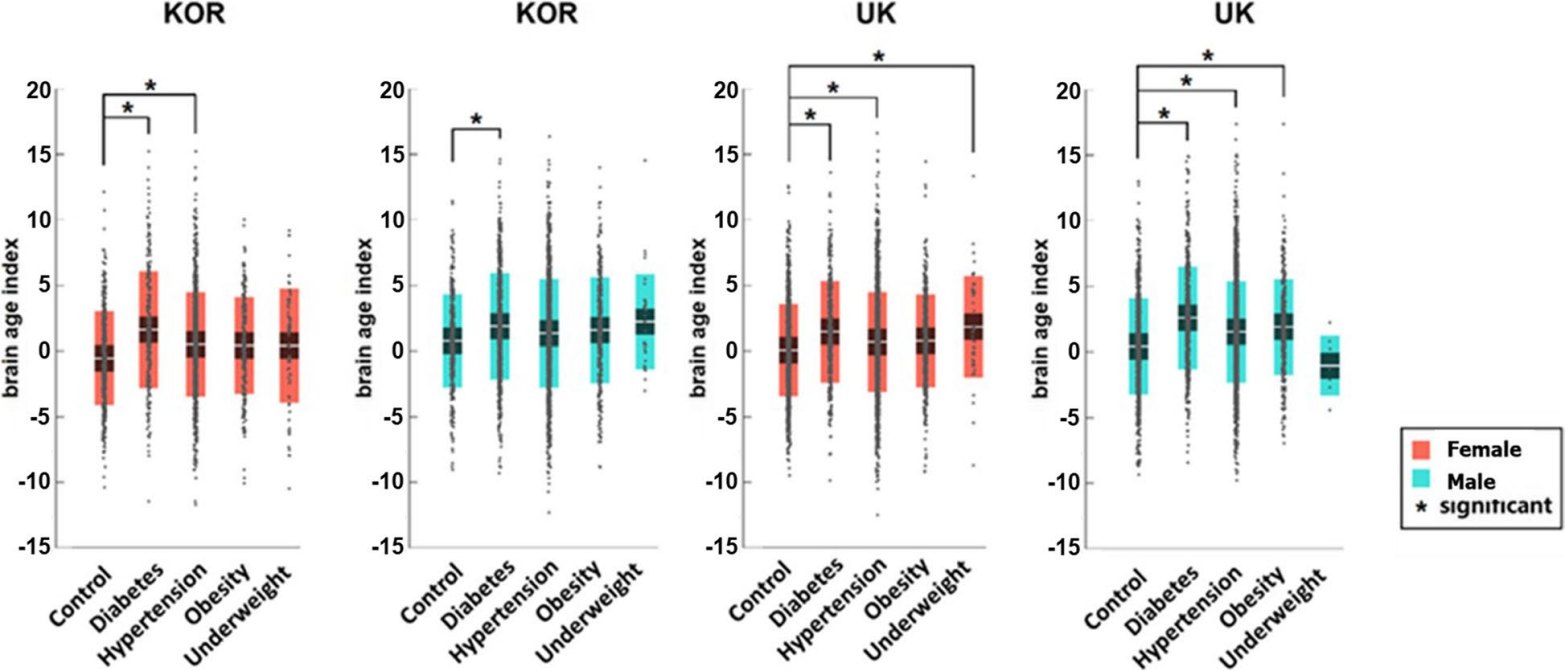
BAI distribution between groups regarding gender and ethnicity for healthy participants and participants with different CMS. BAI = 0 indicates that the chronological age is the same as the predicted brain age, with higher values indicating an older-appearing brain than chronological age. Asterisk symbol (^*^) indicates the following: *q*-values, FDR-corrected *p*-values, are lower than 0.05. BAI, brain age index; Kor, Korea; UK, United Kingdom; CMS, cardiometabolic syndrome

As shown in Fig. 2, DM was associated with increased BAI for all participants, regardless of gender and ethnicity (*q* < 0.001 in the four groups) (Table 2). Hypertension was associated with a significantly higher BAI for all participants (*q* < 0.001), except for Korean males (*q* = 0.309). Obesity significantly increased the BAI for UK males (*q* = 0.004). Being underweight increased the BAI significantly only for UK females (*q* = 0.002).

**Table 2:** Brain age index in controls and four CMS component groups

Ethnicity/gender	CMS	Age	BAI	*β* (SE)	*q*-value^*^
Korean females	Control	61.6 ± 8.1	− 0.67 ± 3.57		
	T2DM	66.9 ± 9.5	1.51 ± 4.44	1.73 (0.25)	< 0.001
	Hypertension	66.0 ± 8.0	0.39 ± 3.97	0.80 (0.20)	< 0.001
	Obesity	64.8 ± 8.5	0.31 ± 3.69	0.26 (0.94)	0.347
	Underweight	62.8 ± 7.6	0.30 ± 4.34	0.77 (0.48)	0.145
Korean males	Control	64.1 ± 8.2	0.70 ± 3.55		
	T2DM	65.5 ± 8.6	1.82 ± 4.05	0.88 (0.19)	< 0.001
	Hypertension	65.3 ± 8.4	1.29 ± 4.13	0.19 (0.18)	0.309
	Obesity	63.4 ± 7.7	1.52 ± 4.03	0.37 (0.23)	0.144
	Underweight	67.5 ± 10.7	2.17 ± 3.63	1.24 (0.72)	0.171
UK females	Control	62.0 ± 7.2	− 0.17 ± 3.50		
	T2DM	64.4 ± 7.4	1.22 ± 3.87	1.08 (0.20)	< 0.001
	Hypertension	65.2 ± 6.6	0.45 ± 3.79	0.49 (0.13)	< 0.001
	Obesity	61.5 ± 6.7	0.53 ± 3.54	0.32 (0.19)	0.088
	Underweight	63.8 ± 7.0	1.60 ± 3.87	1.67 (0.53)	0.002
UK males	Control	61.5 ± 7.5	0.25 ± 3.66		
	T2DM	65.8 ± 6.8	2.42 ± 3.90	1.55 (0.17)	< 0.001
	Hypertension	65.6 ± 6.8	1.35 ± 3.86	0.73 (0.13)	< 0.001
	Obesity	62.9 ± 7.2	1.72 ± 3.63	0.65 (0.22)	0.004
	Underweight	68.0 ± 6.3	− 1.21 ± 2.28	− 1.69 (1.42)	0.235

Values of age and BAI are presented as mean ± standard deviation

*Abbreviations*: *BAI*, brain age index; *CMS*, cardiometabolic syndrome; *T2DM*, type2 diabetes mellitus; *UK*, United Kingdom

^*^q-values, FDR-corrected *p*-values, were obtained using a linear regression analysis with the presence of hypertension, T2DM, obesity, and underweight as covariates in each group (Korean females, Korean males, UK females and UK males)

### Interactive effects of cardiometabolic syndrome components on brain age index in relation to gender and ethnicity

**Fig. 3:**
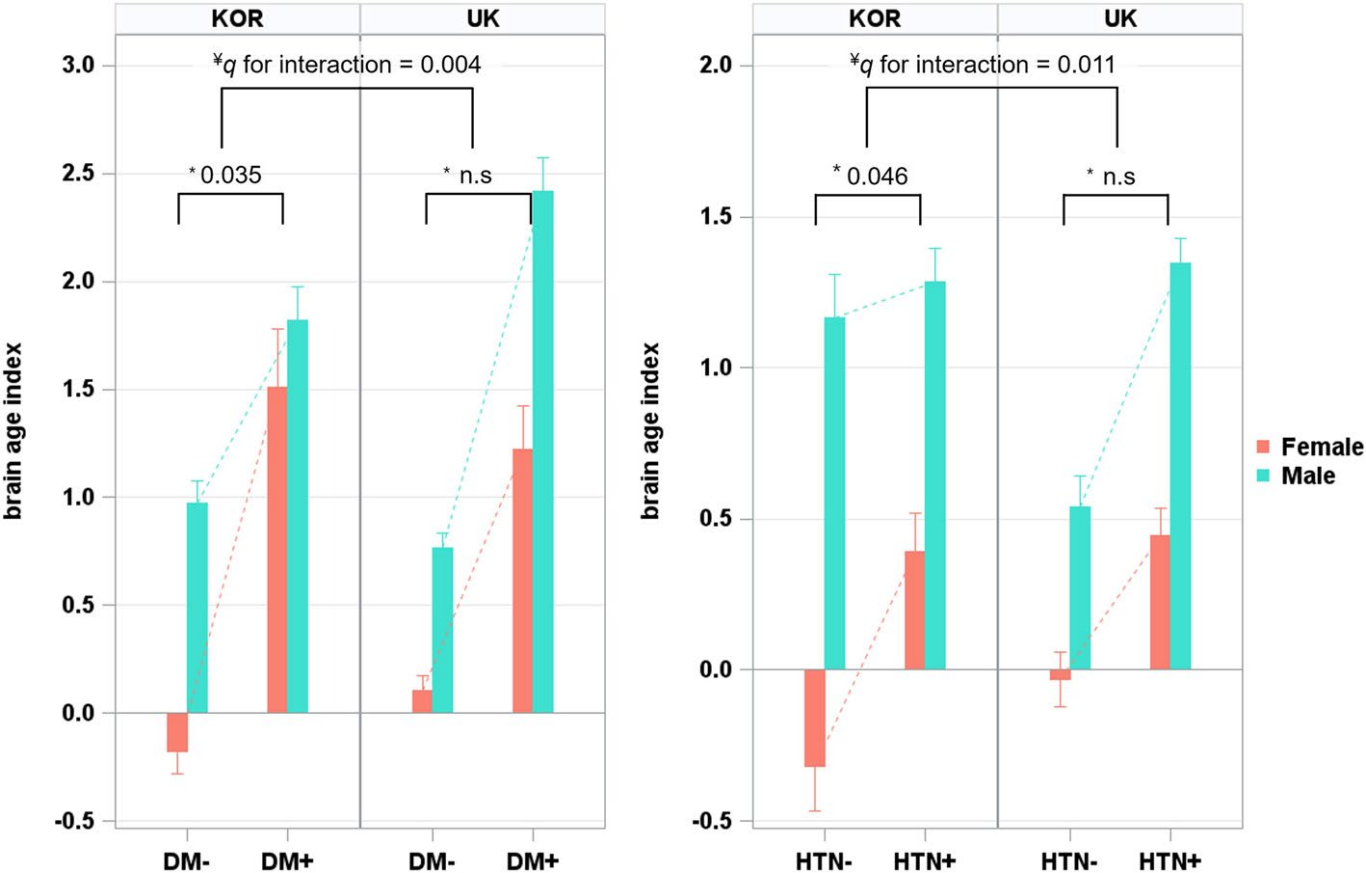
Ethnic- and gender-specific difference in BAI between participants with and without T2DM and HTN. Values depicted in the bar plot represent the mean of BAI, and values depicted in the error bar represent the standard error of mean. BAI = 0 indicates that the chronological age is the same as the predicted brain age, with higher values indicating an older-appearing brain than chronological age. Asterisk (^*^) symbol indicates the following: *q*-values, FDR-corrected *p*-values, were obtained using linear regression analyses with adding each two-way interaction term (the presence of each CMS component*gender) to covariates in Korean and UK populations after controlling for the other CMS components. Yen (^¥^) symbol indicates the following: *q*-values, FDR-corrected *p*-values, were obtained using linear regression analyses with additionally adding each three-way interaction term (the presence of each CMS component*gender*ethnicity) to covariates after controlling for the other CMS components. BAI, brain age index; Kor, Korea; UK, United Kingdom; T2DM, type 2 diabetes mellitus; HTN, hypertension

We also investigated the interaction of the presence of each CMS component and gender with BAI in Koreans and participants from the UK. Among Koreans, there were interactions between T2DM and gender with BAI (*q* = 0.035) and between hypertension and gender with BAI (*q* = 0.046), suggesting that the effects of T2DM and hypertension on BAI were more prominent in females than in males (Table 3, Fig. 3). Among British participants, however, there were no interactions of any CMSs and gender with BAI (*q* range 0.098 to 0.203, Table 3, Fig. 3). In fact, there were interactions between gender and ethnicity for T2DM (*q* = 0.004) and hypertension (*q* = 0.011, Table 3, Fig. 3).

**Table 3:** Interaction effect on the difference in BAI between participants with each CMS and those with control

**Ethnicity**	Two-way interaction (each CMS component*gender)	***β*** ** (SE)**	*q*-value^*^
Korea	T2DM*gender	0.84 (0.32)	0.035
	Hypertension*gender	0.61 (0.27)	0.046
	Obesity*gender	− 0.11 (0.36)	0.770
	Underweight*gender	− 0.82 (0.85)	0.450
UK	T2DM*gender	− 0.52 (0.26)	0.098
	Hypertension*gender	− 0.26 (0.18)	0.203
	Obesity*gender	− 0.37 (0.28)	0.191
	Underweight*gender	3.51 (1.50)	0.076
	Three-way interaction(each CMS component*gender*ethnicity)	***β*** ** (SE)**	***q*-value**^**¥**^
Both	T2DM*gender*ethnicity	1.36 (0.41)	0.004
	Hypertension*gender*ethnicity	0.89 (0.32)	0.011

*Abbreviations*: *BAI*, brain age index; *CMS*, cardiometabolic syndrome; *T2DM*, type2 diabetes mellitus; *UK*, United Kingdom

^*^q-values, FDR-corrected *p*-values, were obtained using linear regression analyses with adding each two-way interaction term (the presence of each CMS component*gender) to covariates in Korean and UK populations after controlling for the other CMS components

^¥^*q*-values, FDR-corrected *p*-values, were obtained using linear regression analyses with additionally adding each three-way interaction term (the presence of each CMS component*gender*ethnicity) to covariates after controlling for the other CMS components

## Discussion

In the present study, we systematically investigated the different effects of CMS on BAI in relation to gender and ethnic differences in a large sample of Korean and UK CU populations. Our major findings are as follows: first, among Koreans, the effects of DM and hypertension on BAI were higher in females than in males. This indicated interaction effects of gender and the presence of T2DM and hypertension on BAI in Korean population. Second, among the UK population, there were no differences in the effects of T2DM and hypertension on BAI between males and females. Overall, there was evidence that ethnicity modified the gender-specific relationship of T2DM and hypertension with BAI. Taken together, our findings suggest that CMS exerts different effects on brain age depending on gender and ethnicity. Therefore, ethnic- and gender-specific prevention strategies may be necessary to protect against accelerated brain aging.

We found that the presence of T2DM and hypertension was associated with a higher BAI regardless of gender and ethnicity, except for hypertension in Korean males. T2DM and hypertension are well-known risk factors for brain atrophy, which is an important indicator of brain age [30]. T2DM may have deleterious effects on the brain via various mechanisms, including cerebrovascular complications, glucose toxicity due to insulin resistance, and chronic inflammation [31]. Similarly, the positive association between hypertension and BAI may be due to several possible mechanisms including cerebral hypoperfusion, micro- and macrovascular damage in white matter, and cerebral microinfarcts [32, 33].

Our first major finding was that Korean females suffered more deleterious effects of T2DM and hypertension on brain age than Korean males. Although the underlying mechanisms for the gender-specific effects of T2DM and hypertension on brain age are not fully understood, our findings might be related to the complex effects of biological and socioeconomic differences [34]. Previous studies have suggested that hypertension exerts worse effects on multiple organs in females than in males. This was attributed to differences in sex hormones. There are stronger associations of hypertension with autonomic dysfunction in females than in males [35]. Similar associations are witnessed in the cases of microalbuminuria [36] and reduction of heart function [37]. In particular, females uniquely experience menopause transition, which might accelerate cardiometabolic syndrome, brain aging, or cognitive impairment via several mechanisms including changes in the availability of estrogen [38], estrogen receptor activity, and/or estrogen-regulated neural networks [39]. Specifically, estrogen deficiency in postmenopausal females leads to inflammatory process and vasoconstriction via the dysfunction of the renin-angiotensin system [40–42]. In fact, a growing body of evidence shows that menopause has a deleterious impact on cognitive function, which may contribute to the higher prevalence of dementia in females than in males [43–47]. Additionally, several studies have shown that females tend to maintain lifestyles that are more favorable for brain health, with overall lower drinking and smoking rates [48–51]. Therefore, our findings might be also related to differences in stress, alcohol consumption, smoking, and dietary habits according to gender.

Our second major finding was that there were interactive effects of the presence of T2DM and hypertension, gender, and ethnicity on BAI. That is, unlike Koreans, there were no differences in the effects of T2DM and hypertension on BAI between males and females in the UK population. A few studies have found that brain age differs depending on ethnicity [52, 53]. However, gender- and ethnicity-specific differences in the effects of T2DM and hypertension on brain age have not been extensively investigated. These differences might be related to the biological and socioeconomic differences between the Korean and UK populations. Previously, a higher frequency of CMS in Korean populations compared to Europeans has been explained by the fact that Asians have higher visceral fat and lower subcutaneous fat than Europeans with the same BMI [54]. This might increase the complication rate of CMS because visceral fat has more deleterious effects on arteriosclerosis and brain health than subcutaneous fat. In fact, Asians are more likely to develop CMS-related complications such as coronary artery disease [55], stroke [56], dementia [57–59], or mortality [60]. Another potential explanation is that there were fewer differences in socioeconomic status and years of education between males and females in the UK than in Korea. Further studies are needed to investigate the pathomechanism to explain gender differences according to ethnicity.

## Limitations

The strengths of our study include a large sample size from two different cohorts, well-balanced clinical demographics between the two cohorts after propensity score matching, and a novel measurement of brain age that is sensitive to neurodegenerative changes in gray and white matter. However, our study had some limitations. First, owing to the cross-sectional study design, the causal or temporal relationship of the effects of CMS on brain aging was not determined. In addition, the study did not have information on exposure time or changes in the status of risk factors. Longitudinal studies are needed to identify whether there are dynamic differences from mid-adulthood to old age in the effects of risk factors on brain aging in elderly CU populations. Second, differences in subject selection methods between the two cohorts may have confounded the ethnic differences. Third, the presence of T2DM and hypertension was determined through the patient history of diagnosis or medications and not through clinical examinations including measurement of systolic blood pressure and fasting glucose. Fourth, obesity was defined using BMI only rather than waist circumstance, which has relevance to central obesity according to the International Diabetes Federation. We also used different criteria for diagnosing obesity according to ethnicity-specific BMI. This was, however, done to abide by a previous consensus on the definition of obesity according to ethnicity [25]. Finally, we did not consider the brain pathology markers of Alzheimer’s disease, lacunes, micro-cortical infarcts, and white matter hyperintensities, which can also be associated with brain age. Further studies are needed to identify the effects of CMS on brain aging in relation to the pathophysiological processes. Despite the aforementioned limitations, our study is the first report to compare the gender- and ethnicity-specific effects of CMS on brain age.

## Conclusions

In the present study, we highlight gender and ethnic differences in the effects of CMS on brain age. Furthermore, our findings suggest that different measures may be needed to prevent accelerated brain aging by CMS in terms of gender and ethnic differences. In conclusion, CMS exerted different effects on brain age according to the gender and ethnicity of the individuals. Our study shows that it is important to control for T2DM and hypertension to prevent brain aging. Since the effects of T2DM and hypertension on brain age were the largest among Korean females, more careful treatment of these CMS components would be more effective to prevent or mitigate fast brain aging in Korean females. Therefore, ethnic- and gender-specific prevention strategies may be needed to protect against accelerated brain aging.

## Data Availability

Anonymized data for our analyses presented in this report are available upon request from the corresponding authors.
